# Superparamagnetic iron oxide nanoparticles drive miR-485-5p inhibition in glioma stem cells by silencing Tie1 expression

**DOI:** 10.7150/ijbs.42887

**Published:** 2020-02-10

**Authors:** Zhiguang Pan, Yongyi Huang, Haiyang Qian, Xiling Du, Wenxing Qin, Te Liu

**Affiliations:** 1 Department of Neurosurgery, Huashan Hospital, Shanghai Medical College, Fudan University, Shanghai 200040, China; 2 School of Environmental and Chemical Engineering, Shanghai University, Shanghai 200444, China; 3 Department of Imaging, Dahua Hospital, Xuhui District, Shanghai 200237, China; 4 School of Life Science and Technology, Tongji University, Shanghai 200092, China; 5 State Key Laboratory of New Drug and Pharmaceutical Process, Shanghai Institute of Pharmaceutical Industry, China State Institute of Pharmaceutical Industry, Shanghai 200437, China; 6 Department of medical oncology, Shanghai Changzheng hospital, Second Military Medical University, Shanghai 200003, China; 7 Shanghai Geriatric Institute of Chinese Medicine, Shanghai University of Traditional Chinese Medicine, Shanghai 200031, China

**Keywords:** Superparamagnetic iron oxide nanoparticles (SPIONs), microRNA, glioma stem cells, Tie1

## Abstract

Gliomas are highly malignant nervous system tumours. Studies shown that cancer stem cells are one of the main reasons underlying recurrence, metastasis, and poor prognosis in glioma cases. Our previous studies have found that superparamagnetic iron oxide nanoparticles (SPIONs) can act as nucleic acid carriers to drive intracellular overexpression of these nucleic acids. In this study, CD44+/CD133+ glioma stem cells (HuGSCs) were first isolated from surgically resected tissues from patients. qPCR and western blot results showed that Tie1 expression in HuGSCs was significantly higher thanexpression in CD44-/CD133- glioma cells. Bioinformatic analysis and luciferase reporter assays showed that miR-485-5p binds to specific loci on the 3′-UTR of *Tie1* mRNA to inhibit Tie1 expression. Subsequently, miR-485-5p/miR-mut and SPION complexes were transfected into HuGSCs. Transmission electron microscopy showed that a highly dense metallic electron cloud is present in HuGSCs. At the same time, *in vivo* and *in vitro* studies showed that miR-485-5p@SPIONs can significantly inhibit HuGSC proliferation, invasion, tumourigenicity, and angiogenesis. In-depth analysis showed that Tie1 interacts with neuronal growth factors such as FGF2, BDNF, GDNF, and GFAP. qPCR and western blot results showed that in miR-485-5p@SPIONs-HuGSCs, the expression levels of Tie1 and stem cell markers (Oct4, Sox2, Nanog, CD44, and CD133), and even FGF2, BDNF, GDNF, and GFAP were significantly lower than thelevels in the control group (miR-mut@SPIONs-HuGSCs). Therefore, this study showedthat Tie1 is an important factor that maintains glioma stem cell activity. SPIONs drive miR-485-5p overexpression in cells and inhibit endogenous Tie1 expression to downregulate the protein expression levels of Fgf2/GDNF/GFAP/BDNF and significantly weaken the *in vivo* and *in vitro* viability of gliomas.

## Introduction

More and more experiments have shown the presence of a unique cell subpopulation in tumour tissues that has strong proliferation and invasion capacities, is resistant to multiple chemotherapeutic agents, and express many markers in embryonic stem cells. These cells are known as cancer stem cells 1-4. Studies have found that cancer stem cells only account for 0.3-0.7% of all cancer cells, but they are one of the key factors that cause chemotherapy failure, metastasis, and recurrence. Gliomas are the most common primary central nervous system tumours. Studies have found that cancer stem cells are also present in gliomas 1-7. Currently recognised specific markers for human glioma stem cells (HuGSCs) include CD133, integrin α6, CD171 (L1CAM), CD15, nestin, and CD44 1, 5-7. Although HuGSCs have been discovered, the reasons for their high degree of malignancy are still not completely clear. On the other hand, microRNAs are important factors that regulate growth and development, tumourigenesis, and tumour progression, and also have important regulatory effects on glioma viability 7-9.

Tie1 is a member of the receptor tyrosine kinase Tie family 10-13. Tie1 is structurally similar to its homolog Tie2, but Tie1 has no known ligands, in contrast to Tie2. Therefore, Tie1 is an orphan receptor 10-13. The key function of Tie1 is to form a heterodimer with Tie2 on the cell surface, which regulates Tie2 signal transduction 10-13. The effects of Tie1-Tie2 interactions depend on the environment. Heterodimerization can promote or inhibit downstream Tie2 signal transduction and depending on the local expression level of Tie2 10-13. Studies have shown that Tie1 is associated with angiogenesis, vascular maturation, tissue remodelling, and inflammation. Recent studies have also shown that elevated Tie1 expression is intimately associated with atherosclerosis and the stemness of cancer stem cells 12, 14-17. However, there have beenno reports so far on the relationship between Tie1 and HuGSCs.

Magnetic nanomaterials are a type of nanomaterial generally used to describe materials with particle sizes of 0-100nm, that are composed of iron, cobalt, nickel, and their alloys that can directly or indirectly produce magnetism 7, 18-20. In recent years, there has been growing attention on the use of magnetic nanomaterials as gene carriers. Superparamagnetism usually occurs when the size of magnetic nanoparticles is less than 20 mm 7, 18-20. Due to the controllability of their traits, good stability, and easy modification, superparamagnetic iron oxide nanoparticles (SPIONs) have currently become a research hotspot for gene carriers. After SPIONs bind to plasmid DNA and siRNA/microRNA, nucleic acids can be transfected into mammalian cells under the effects of an external magnetic field. Magnetic adsorption is used to overcome intracellular and extracellular barriers, increase local DNA/RNA concentration, and increase transfection efficiency 7, 18-20. Further research found that surface modification with polyethylenimine (PEI), dendrimers, glucose, chitosan, and other cationic polymers or cationic lipids can improve the transfection efficiency of nanomaterials and nucleic acids^7^, ^18^-^20^. Our previous studies have found that SPIONs can efficiently drive the intracellular transport and expression of siRNA or microRNA to inhibit the proliferation and invasion of glioma stem cells or endometrial cancer stem cells 7, 18-20.

In summary, we hypothesise that Tie1 has important regulatory effects on the stemness and malignancy of HuGSCs. Therefore, we employed bioinformatics to predict microRNAs that regulate Tie1 in this study, and constructed microRNA@SPION complexes to increase their delivery and expression efficiency in HuGSCs. This method was used to validate differences in the proliferation, invasion, and tumourigenicity of HuGSCs before and after Tie1 downregulation.

## Materials and Methods

### Isolation and incubation of primary CD44+/CD133+ HuGSCs

According to the privious study 6, 7, primary CD44+/CD133+ HuGSCs were isolated from tumour tissues surgically resected from 6 glioma patients in the Department of Neurosurgery at the Shanghai Huashan Hospital (The median age of these populations was 45 years (45.5±7.0); poorly differentiated variant star glioma (stage III)) . Weight of approximation 400mg glioma tissues were digested with 0.25% trypsin under sterile conditions. Approximation 1x10^6^ cells were collected via centrifugation of the cell suspension, followed by the addition of 0.5 mL of ice-cold sterile PBS (HyClone). Five microliters of anti-human CD133-PE antibody (eBioscience, Inc., San Diego, CA, USA) and anti-human CD44-FITC antibody (eBioscience) were added into approximation 1x10^6^ cells at the same time to a final concentration of 0.01 mg/mL and mixed well. The cells were incubated at 4 °C in the dark for 30 minutes. After completion of the reaction, the cells were washed twice with ice-cold PBS and then CD133^+^ human primary GSCs were sorted and enriched using flow cytometry (BD FACS Aria, BD Bioscience, CA, USA). After Sorting, approximation 2x10^4^ CD44+/CD133+ cells could be isolated. The concentration of the cells was adjusted to 1000 cells/mL, and the cells were seeded into non-adherent spherical clusters. The cells were incubated with DMEM:F12 (HyClone) medium containing 10 ng/mL basic fibroblast growth factor (bFGF), 10 ng/mL epidermal growth factor (EGF), 5 μg/mL insulin and 0.5% bovine serum albumin (BSA) (all from Sigma-Aldrich, St Louis, MO, USA). The cells were cultured to the third generation and then used for the subsequent experiments.

### Induction ofthe transfection of microRNA into cells using SPIONs

According to the privious study 6, 7, 18, 19, SPIONs were purchased from NOVOBIO (NOVOBIO Biotechnology Co., Ltd., Shanghai, China). According to the manufacturer's instructions and previously published methods, 5 μl of 0.2 mM SPIONs was thoroughly mixed with 5 μl of 1.0 or 10 μM miR-485-5p oligonucleotide RNA (CUUAAGUAGUGCCGGUCGGAGA) (Sigma-Aldrich) or miR-mut (CUUAAGUAGUGCCGGUgccucc), vortexed for 10 s, and then maintained at room temperature for 20 min. A total of10 μl of the microRNA@SPIONs mixture was then combined with 90 μl of DMEM:F12 (1:1) serum-free medium, added to 1 x 10^4^ cells/ml, and incubated for 72 h at 37 °C in 5% CO_2_.

### Luciferase report assay

All cells were seeded into 24-well cell culture plates at a density of 3 × 10^4^ cells/well. The Lipofectamine 2000 Reagent was used to transfect the cells in the respective groups with 400 ng of miR-485-5p@SPIONs or miR-mut@SPIONs and 20 ng of pSiCHECK2-Tie1-3UTR or pSiCHECK2-Tie1-mut (NOVOBIO Biotechnology Co., Ltd., Shanghai, China). At 48 h after transfection, the dual-luciferase reporter assay system (Beyotime Biotechnology Co., Ltd., Zhejiang, China) was used to detect the luciferase activity in each group.

### Quantitative real-time PCR

Quantitative real-time PCR (qPCR) was performed according to the manufacturer's protocol of the miRcute miRNA qPCR Detection kit (TIANGEN Biotech, Shanghai, China). Briefly, the reaction comprised: 10 μL of 2 × miRcute Plus miRNA Premix (with SYBR), 1 × 1 μL (10 μM) of forward primer, 1 × 1 μL (10 μM) of reverse primer, 4 μL of first strand cDNA from miRNA, and 4 μL of deionized water. The following reactions were carried out in a real-time PCR machine: 40 cycles of 95 °C for 15 min, 94 °C for 20 s, and 60 °C for 34 s. The fluorescence values were recorded. The sequences of the qPCR primers were as: Fgf2-F: AGAAGAGCGACCCTCACATCA; Fgf2-R: CGGTTAGCACACACTCCTTTG; Gdnf-F: GGCAGTGCTTCCTAGAAGAGA; Gdnf-R: AAGACACAACCCCGGTTTTTG; Bdnf-F: CTACGAGACCAAGTGCAATCC; Bdnf-R: AATCGCCAGCCAATTCTCTTT; Gfap-F: AGGTCCATGTGGAGCTTGAC; Gfap-R: GCCATTGCCTCATACTGCGT; Oct4-F: CAGTGCCCGAAACCCACAC; Oct4-R: GGAGACCCAGCAGCCTCAAA; Sox2-F: AACCCCAAGATGCACAACTC; Sox2-R: GCTTAGCCTCGTCGATGAAC; Nanog-F: GATTTGTGGGCCTGAAGAAA; Nanog-R: ATGGAGGAGGGAAGAGGAGA; Cd44-F: CTGCCGCTTTGCAGGTGTA; Cd44-R: CATTGTGGGCAAGGTGCTATT; Cd133-F: AGTCGGAAACTGGCAGATAGC; Cd133-R: GGTAGTGTTGTACTGGGCCAAT; miR-485-5p-F: AGAGGCTGGCCGTGATGAATTC; miR-485-5p-R: GCTGTCAACGATACGCTACCTA; miR-188-5p-F: CTCCCACATGCAGGGTTTGCA; miR-188-5p-R: GCTGTCAACGATACGCTACCTA; miR-486-3p-F: CGGGGCAGCTCAGTACAGGAT; miR-486-3p-R: GCTGTCAACGATACGCTACCTA; miR-362-3p-F: AACACACCTATTCAAGGATTCA; miR-362-3p-R: GCTGTCAACGATACGCTACCTA; miR-329-3p-F: AACACACCTGGTTAACCTCTTT; miR-329-3p-R: GCTGTCAACGATACGCTACCTA; miR-194-3p-F: CCAGTGGGGCTGCTGTTATCTG; miR-194-3p-R: GCTGTCAACGATACGCTACCTA; miR-939-5p-F: TGGGGAGCTGAGGCTCTGGGGGTG; miR-939-5p-R: GCTGTCAACGATACGCTACCTA; miR-28-5p-F: AAGGAGCTCACAGTCTATTGAG; miR-28-5p-R: GCTGTCAACGATACGCTACCTA; miR-708-5p-F: AAGGAGCTTACAATCTAGCTGGG; miR-708-5p-R: GCTGTCAACGATACGCTACCTA; 18S rRNA-F: CAGCCACCCGAGATTGAGCA; 18S rRNA-R: TAGTAGCGACGGGCGGTGTG.

### Transmission electron microscopy analysis

The samples were fixed and embedded according to procedures described previously 6, 7, 19. Tissue samples were first fixed in 1% glutaraldehyde (Sigma-Aldrich, St. Louis, USA) for 4 hand then fixed in 1% osmium tetroxide for 1 h, followed by dehydration in acetone; finally, the samples were embeddedin resin 12 (Ted Pella, USA).Ultra-thin slices (thickness of 70nm) of the samples were generated and were attached to a copper mesh. The sections were stained with 1% uranium acetate (Sigma-Aldrich, St. Louis, USA) and 1% lead citrate (Sigma-Aldrich, St. Louis, US) and were then imaged with a JEM-1230 transmission electron microscope (JEOL, Japan).

### Cell proliferation assay

Cells were seeded at 2 × 10^3^ per well in 96-well plates and cultured in DMEM (Gibco, Gaithersburg, MD, USA) supplemented with 10% fetal bovine serum (FBS) at 37 °C under 5% CO_2_ until 85% confluence. 3-(4,5-dimethylthiazol-2-yl)-2,5-diphenyltetrazolium bromide (MTT; Sigma-Aldrich) (5 mg/mL) was added at different time points and incubated for a further 4 h. The reaction was terminated by adding 150 μL/well dimethyl sulfoxide (Sigma-Aldrich). Cells were lysed for 15 min, and the plates were gently shaken for 5 min. Absorbance at 490 nm was determined using a Model 680 Microplate Reader (Bio-Rad, MA, USA).

### Northern blot

Northern blotting was performed as previously described 21. Briefly, total RNA was extracted from all groups of cells using a Trizol kit. Following quantification, 20 μg of high-quality total RNA was subjected to gel electrophoresis on a 7.5 M urea-12% formaldehyde (PAA) denaturing gel. Afterwards, the RNA was transferred to a Hybond N+ nylon membrane (Amersham, Freiburg, Germany). The membrane was cross-linked under 1200 mjoule/cm2 of UV for 30 s. An antisense DNA probe against miR-485-5p was used for hybridization to detect the expression status of miR-485-5p (5'-AGAGGCTGGCCGTGATGAATTC-3'). After hybridization and washing, the membrane was exposed to Kodak XAR-5 films (Sigma-Aldrich) for 20-40 h. As a positive control, the human U6 snRNA probe (5-GCAGGGGCCATGCTAATCTTCTCTGTATCG-3) was used for hybridization in all membranes. The exposure time for the U6 snRNA probe was maintained between 15 and 30 min. The gray levels of Northern blotting hybridization bands were quantified by using ImageJ software. To determine relative levels of hybridisation signal, the formula was used as follows: Target hybridisation signal gray value/U6 snRNA gray value. The hybridisation signal levels were calibrated based on levels of U6 snRNA.

### Western blot

Western blotting was performed as previously described 18, 21. Briefly, total protein from each group of cells was subjected to SDS-PAGE on a 12% denaturing gel. Afterwards, the proteins were transferred to a polyvinylidene fluoride (PVDF) membrane (Millipore, MA, USA). After blocking and washing the membrane, primary antibodies (Table 1) were added and the membrane was incubated at 37 °C for 15 min. After extensive washing, secondary antibodies (Table 1) were added and incubated at 37 °C for 45 min. The membrane was subjected to four 14 min washes with Tris-buffered saline-Tween 20 (TBST) at room temperature. The membrane was then developed using an enhanced chemiluminescence (ECL) kit (Pierce Biotechnology, MA, USA) and exposed to X-ray film (Sigma-Aldrich) for visualization. The gray levels of western blotting protein band were quantified by using ImageJ software (Rasband, W.S., ImageJ, U. S. National Institutes of Health, Bethesda, MD, USA). To determine relative expression levels of target proteins, the formula was used as follows: (Experiment group_target protein gray level value/Experiment group_GAPDH protein gray level)/(Control group_target protein gray level value/Control group_GAPDH protein gray level) value. The protein levels were calibrated based on levels of GAPDH.

### In vivo xenograft experiments

According to previous research 7, 19, briefly, after transfection with plasmids, 1 × 10^5^ cells/mL from each group at logarithmic growth phase were harvested and inoculated subcutaneously into BALB/C^nu/nu^ mice. Each group comprised six mice (6-8 week-old female BALB/C^nu/nu^ mice were provided by the Experimental Animal Centre of Fudan University). After 10 weeks of monitoring, the mice were sacrificed, and the tumours were removed. The tumours were weighed, and the volumes were calculated using the following formula: Tumour volume (mm^3^) = (ab2)/2 (a: the longest axis (mm), b: the shortest axis (mm)). All the animal experiments were conducted in accordance with the guidelines of the NIH for the care and use of laboratory animals. The study protocol was also approved by the Committee on the Use of Live Animals in Teaching and Research, Shanghai Geriatric Institute of Chinese Medicine, Shanghai, China.

### Hematoxylin and eosin staining

Tissue samples were fixed in 4% paraformaldehyde, dehydrated, and embedded in paraffin. The paraffin-embedded tissues were cut into 4-μm sections using a microtome, and the sections were affixed onto glass slides. Subsequently, the sections were dewaxed using xylene and subjected to dehydration in an ethanol gradient. The sections were stained with hematoxylin (H) for 5 min at room temperature, and then 1% ethanol was added for 30 s for differentiation. Afterwards, aqueous ammonia was added for 1 min for blueing, followed by rinsing in distilled water for 5 min. Subsequently, the sections were stained with eosin (E) for 2 min at room temperature and then rinsed with distilled water for 2 min. Then, decolorization over an ethanol gradient was performed, and xylene was added for 2 min for clearing. Finally, the sections were sealed and mounted with neutral resin.

### Immunofluorescence staining

Briefly, fresh tissues were immersed in 4% paraformaldehyde (Sigma-Aldrich) for fixation at room temperature for 30 min. The tissues were then dehydrated in an ethanol gradient, embedded in paraffin, sectioned (thickness: 6 μm), and immersed in xylene for dewaxing. Tissue sections were blocked with immunohistochemical blocking solution (Beyotime Biotechnology Co., Ltd., Zhejiang, China) at 37 °C for 30 min. The blocking solution was then discarded, and the sections were washed three times at room temperature for 5 min each with immunohistochemical washing solution (Beyotime Biotechnology). Then, primary antibodies (Table 1) were added and incubated at 37 °C for 45 min. After incubation, the antibody solution was discarded, and the sections were washed three times at room temperature for 5 min each with immunohistochemical washing solution (Beyotime Biotechnology). Then, secondary antibodies (Table 1) were added and the tissues were incubated at 37 °C for 45 min. After incubation, the antibody solution was discarded, and the sections were washed three times at room temperature for 5 min each with immunohistochemical washing solution (Beyotime Biotechnology). Finally, immunofluorescence blocking solution (Sigma-Aldrich) was added, and the sections were mounted.

### Propidium iodide staining and flow cytometry

Propidium iodide (PI) staining and flow cytometry were performed as previously described 6, 18. Briefly, 5 × 10^5^ cells/ml were harvested and fixed in 1 mL of ice-cold 70% ethanol for 48 h. The cells were centrifuged at 1500 r/min for 5 min at 4 °C. The cell pellets were collected, treated with PI staining solution (Sigma Chemicals), and incubated in the dark at 4 °C for 30 min. A flow cytometer (BD FACSAria, Carlsbad, CA, USA) was used to determine the cell cycle distribution of each group of cells, and data analyses were performed using the CellQuest software.

### Transwell migration assay

A total of 200 μL of serum-free cell culture medium containing 2×10^3^/mL cells was seeded into the top chamber of a Transwell chamber with an 8.0-μm pore size. A total of 600 μl of complete medium containing 10% FBS was inoculated into the lower chamber of the Transwell chamber. The cells were cultured at 37 °C with 5% CO_2_ for 48 h. The cells adhering to the membrane surface were fixed with 4% paraformaldehyde at room temperature for 30 min and stained with 4,6-diamidino-2-phenylindole (DAPI, Sigma-Aldrich) for 10 min. Three non-overlapping fields under the microscope were chosen to calculate the total number of cells.

### Statistical analysis

Each experiment was performed as least three times; data are presented as mean ± the standard error (SE) where applicable. Differences were evaluated using Student's t-tests. P values < 0.05 were considered statistically significant.

## Results

### miR-485-5p negatively regulates Tie1 expression in HuGSCs

**Fig 1:**
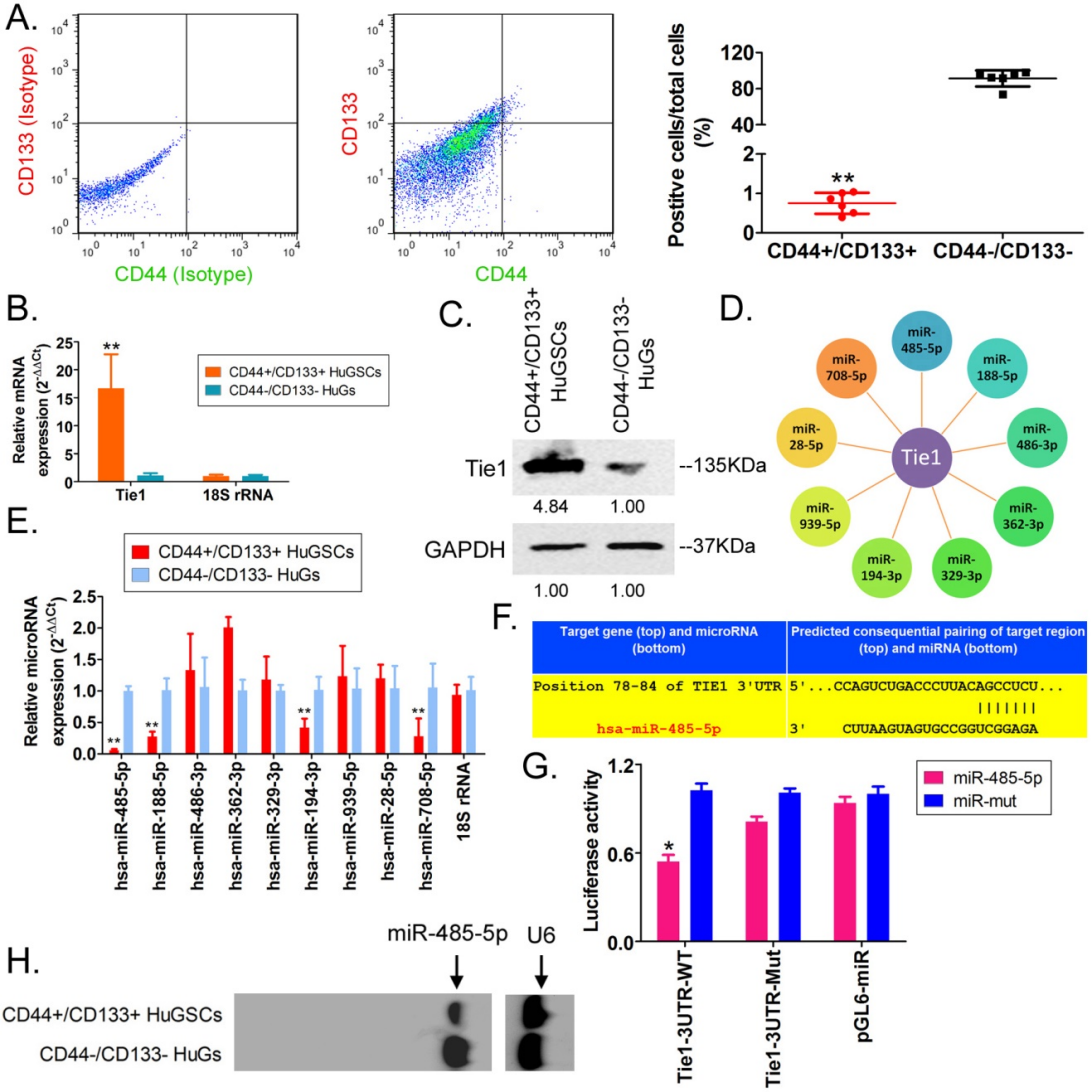
miR-485-5p negatively regulates Tie1 expression in human glioma stem cells (HuGSCs). (A) Flow cytometry results showed that the proportion of CD44+/CD133+ HuGSCs is significantly lower than that of CD44-/CD133- HuGs. ** *p*<0.01 vs. CD44-/CD133- HuGs group. t test. n=6. (B) qPCR results showed that Tie1 is significantly expressed in CD44+/CD133+ HuGSCs. **p<0.01 vs. CD44-/CD133- HuGs group. t test. n=6. (C) Western blot results showed that Tie1 is significantly expressed in CD44+/CD133+ HuGSCs. **p<0.01 vs. CD44-/CD133- HuGs group. t test. n=6. (D) Bioinformatics software predicted that 9 microRNAs target and regulate Tie1. (E) qPCR results showed that 4 microRNAs are significantly expressed in CD44+/CD133+ HuGSCs. **p<0.01 vs. CD44-/CD133- HuGs group. t test. n=3. (F) Northern blot results showed that the miR-485-5p hybridisation signal in CD44+/CD133+ HuGSCs is significantly weakened. (G) Bioinformatics software predicted that miR-485-5p is complementary to the 3′-UTR of Tie1 mRNA. (H) Reported luciferase assay results showed that miR-485-5p can significantly inhibit the activity of luciferase containing specific loci of the Tie1 3′-UTR region. *p<0.05 vs. miR-mut group. t test. n=3.

First, we employed antibody labelling and flow cytometry to enrich CD44+/CD133+ HuGSCs from tumour tissues that were surgically resected from six patients with gliomas. Statistical analysis showed that CD44+/CD133+ HuGSCs accounted for 1% of all tumour cells (Figure 1A). qPCR and western blot results showed that Tie1 expression in CD44+/CD133+ HuGSCs was significantly higher than in CD44-/CD133- HuGs (Figures 1B, C), suggesting that Tie1 expression is positively correlated with the degree of malignancy. Bioinformatics analysis (TargetScanHuman tools Version 7.2; http://www.targetscan.org/vert_72/) showed that Tie1 may be a target of 9 microRNAs (Figure 1D). qPCR results showed that only miR-485-5p, miR-188-5p, miR-194-3p, and miR-708-5p were downregulated in CD44+/CD133+ HuGSCs, amongwhich the downregulation of miR-485-5p was the most significant (Figure 1E). Nucleic acid alignment analysis found that mature miR-485-5p completely complements and pairs with seven bases at a specific locus (+78~+84bp) on Tie1, suggesting that Tie1 may be a target of miR-485-5p (Figure 1F). Luciferase reporter assay results showed that WT miR-485-5p overexpression causes a significant reduction in luciferase activity when it contains the specific 3′-UTR sequence of WT Tie1 while other combinations do not affect luciferase expression (Figure 1G). In addition, northern blot results also showed that the miR-485-5p hybridisation signal in CD44+/CD133+ HuGSCs was significantly lower thanthat in CD44-/CD133- HuGs (Figure 1H). The above results showed that Tie1 is highly expressed in glioma stem cells and is a target of negative regulation by miR-485-5p.

### microRNA@SPIONS can accumulate in HuGSCs and downregulate endogenous Tie1 expression

**Fig 2:**
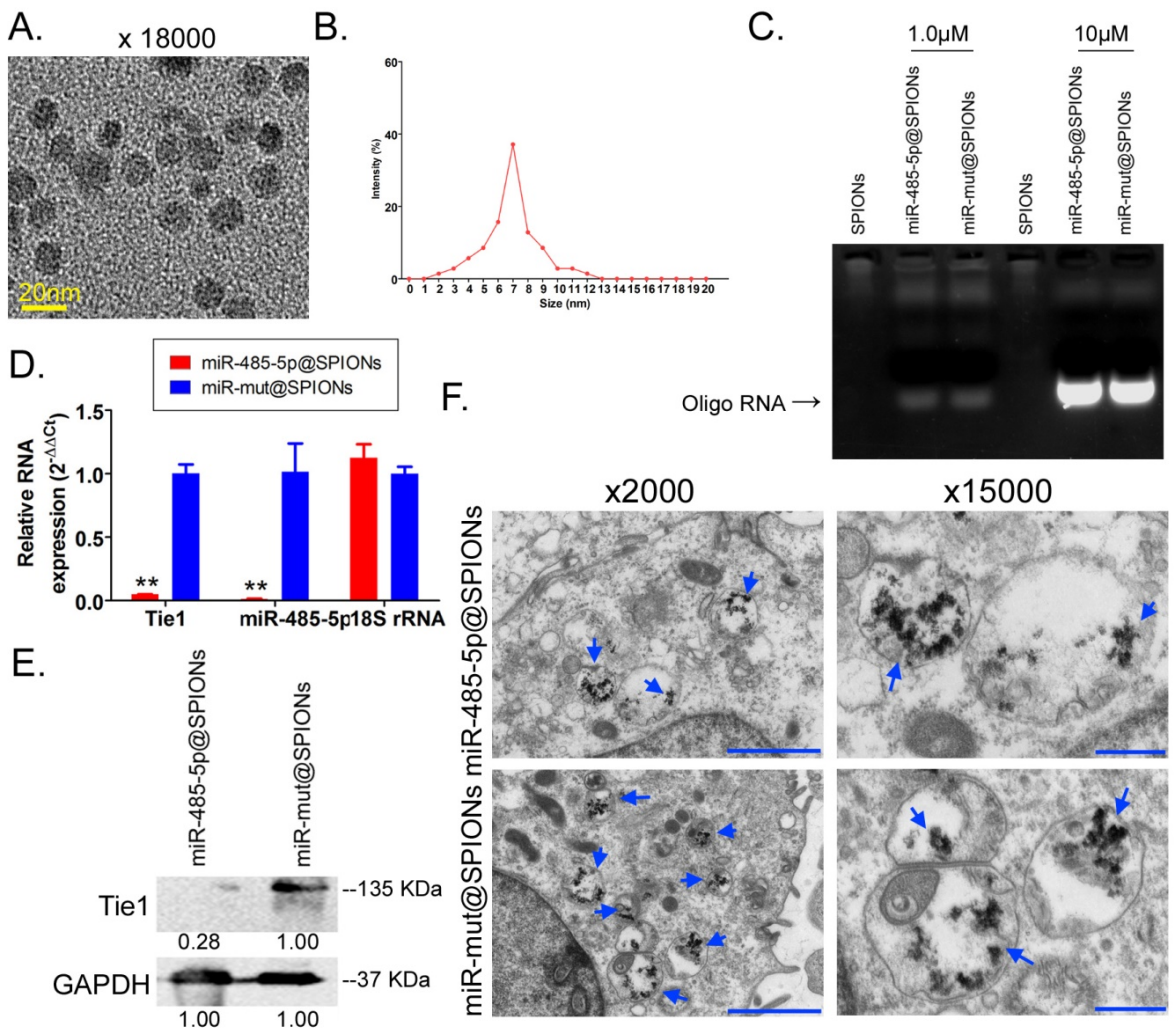
miR-485-5p@SPIONs downregulate Tie1 expression in human glioma stem cells HuGSCs. (A) SPION particles under the transmission electron microscope. (B)Particle size analysis found that the size of the inner core of SPIONs ranged from 2-13 nm. (C) Agarose gel electrophoresis showed that miR-485-5p can bind to SPIONs. (D) qPCR results showed that Tie1 expression is significantly lower in miR-485-5p@SPIONs-HuGSCs than in miR-mut@SPIONs-HuGSCs. **p<0.01 vs. miR-mut@SPIONs-HuGSCs group. t test. n=3. (E) Western blot results showed that Tie1 expression level is significantly lower in miR-485-5p@SPIONs-HuGSCs than in miR-mut@SPIONs-HuGSCs. **p<0.01 vs. miR-mut@SPIONs-HuGSCs group. t test. n=3. (F) Transmission electron microscopy results showed that a large number of highly dense electron cloud substances (SPIONs) aggregated in the microvesicles in the cytoplasm of HuGSCs.

Transmission electron microscopy analysis found that the microstructure of SPIONs takes the form of spherical particles (Figure 2A), with an inner core size of 2-13 nm (Figure 2B). After the surfaces of SPIONs underwent PEI modification, they could efficiently bind to miR-485-5p-expressing plasmids to form a “nucleic acid-nanoparticle” complex (Figure 2C). Different concentrations of miR-485-5p or miR-mut oligonucleic acid chains were used for *in vitro* cross-linking with SPIONs. Following that, agarose gel electrophoresis was used to analyse the cross-linking status of the two reactants. The gel results showed that different concentrations (0.1µM and 10µM) of miR-485-5p or miR-mut oligonucleic acid chains could successfully cross-link with SPIONs (0.2 mM) *in vitro* (Figure 2D). Therefore, a microRNA concentration of 10µM was selected for subsequent experiments. When the surfaces of SPIONs were loaded with large amounts of microRNA, the hydrodynamic dimensions of SPION nanoparticles significantly increased (48.0 nm before loading; 161.5 nm after loading). As oligonucleic acids are negatively charged, the Zeta potential significantly decreased after adsorption (+30.5 mV before loading; +26.3 mV after loading), but a strong positive charge was still maintained, enabling nanoparticles to have good stability, and aggregation and deposition did not occur. microRNA@SPIONs were added to the culture medium of HuGSCs. Transmission electron microscopy showed that a large number of highly dense electron cloud substances aggregated in the microvesicles in the cytoplasm of HuGSCs, and these were speculated to be HuGSC particles (Figure 2E). In addition, qPCR and western blot results all showed that Tie1 expression is significantly lower in miR-485-5p@SPIONs-HuGSCs compared with expression in miR-mut@SPIONs-HuGSCs (Figures 2F, G). The above results showed that microRNA@SPIONs can accumulate in HuGSCs and downregulate endogenous Tie1 expression.

### miR-485-5p@SPIONs significantly inhibit the *in vitro* viability of HuGSCs

**Fig 3:**
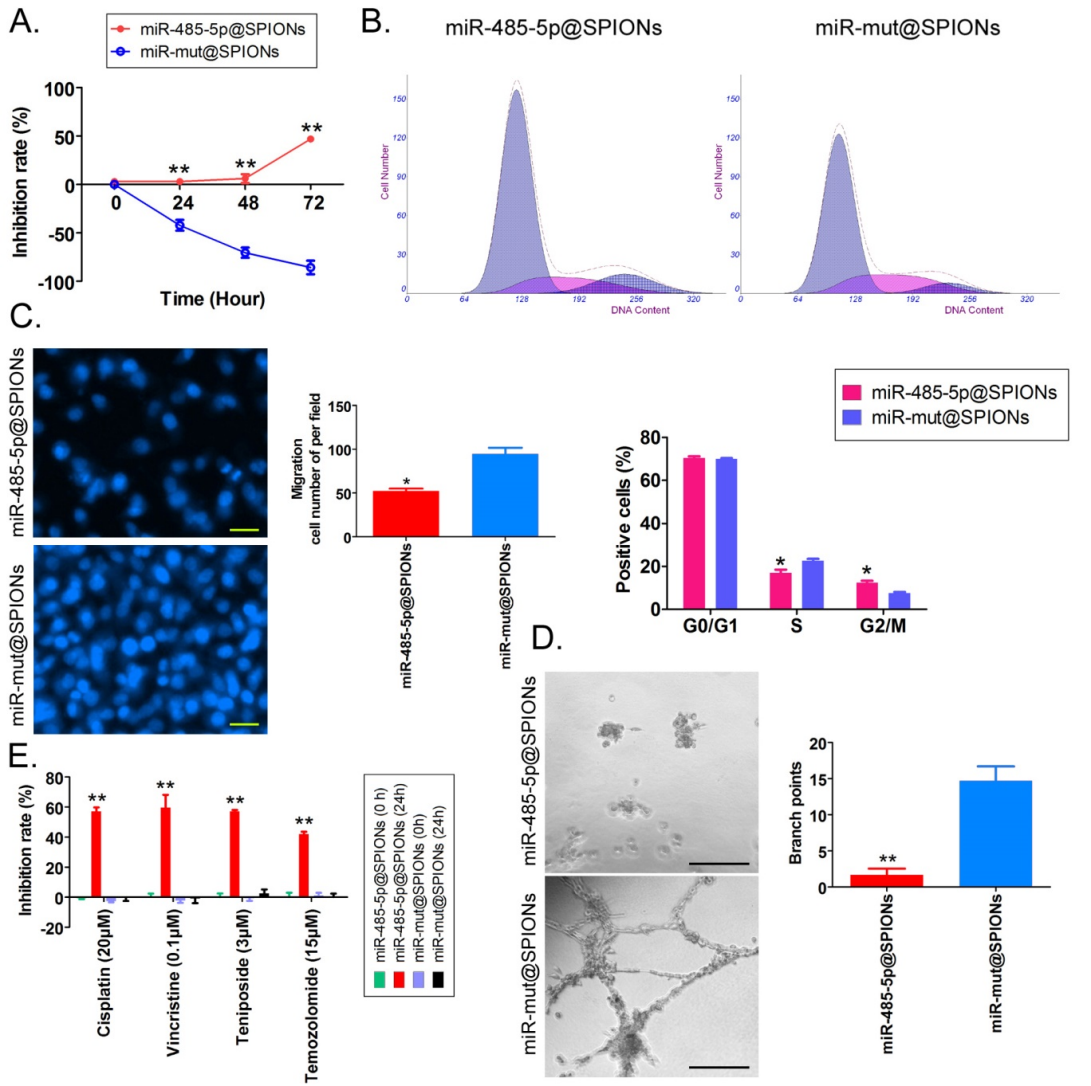
miR-485-5p@SPIONS inhibit the *in vitro* viability of human glioma stem cells (HuGSCs). (A) MTT results showed that proliferation inhibition in miR-485-5p@SPIONs-HuGSCs increases gradually and is time-dependent. **p<0.01 vs. miR-mut@SPIONs-HuGSCs group. t test. n=3. (B) Flow cytometry results showed that the proportion of miR-485-5p@SPIONs-HuGSCs in the G2/M phase was significantly higher than in the control group, while the proportion of cells in the S phase was significantly lower than that in the control group. **p<0.01 vs. miR-mut@SPIONs-HuGSCs group. t test. n=3. (C) Transwell invasion assays showed that the number of migrated miR-485-5p@SPIONs-HuGSCs was significantly lower than those in the control group. *p<0.05 vs. miR-mut@SPIONs-HuGSCs group. t test. n=3. Scale bar = 30μm. (D) The extracellular matrix migration and capillary formation capacities of miR-485-5p@SPIONs-HUVECs were significantly lower than that of miR-mut@SPIONs-HUVECs. *p<0.05 vs. miR-mut@SPIONs-HUVECs group. t test. n=3. Scale bar = 30μm. (E) MTT assay results showed that proliferation inhibition by various chemotherapeutic agents (IC_50_ concentration) is significantly higher in miR-485-5p@SPIONs-HuGSCs than in miR-mut@SPIONs-HuGSCs. **p<0.01 vs. miR-mut@SPIONs-HuGSCs group. t test. n=3.

First, MTT results showed that proliferation inhibition in miR-485-5p@SPIONs-HuGSCs gradually increased 24 hours after microRNA@SPIONs were transfected into cells, and this was significantly higher than inhibition in the miR-mut@SPIONs-HuGSCs (control group). In addition, proliferation inhibition rates were significantly time dependent (Figure 3A). Flow cytometry results showed that the proportion of miR-485-5p@SPIONs-HuGSCs in the G2/M phase was significantly higher than thatin the control group, while the proportion of cells in the S phase was significantly lower than thatin the control group 48 hours after microRNA@SPIONs were transfected into cells (Figure 3B), suggesting that significant cell cycle arrest at the G2/M phase occurred in miR-485-5p@SPIONs-HuGSCs. At the same time, Transwell invasion assays showed that the number of migrated miR-485-5p@SPIONs-HuGSCs was significantly lower than that of the control group 48 hours after microRNA@SPIONs were transfected into cells (Figure 3C). After miR-485-5p@SPIONs were transfected into human umbilical vein endothelial cells (HUVECS), their migration and capillary formation capacities in the extracellular matrix were significantly lower than that of cells transfected with miR-mut@SPIONs (Figure 3D). In addition, MTT assays were used to measure the proliferation inhibition effects of common chemotherapeutic agents (IC_50_ concentration). The experimental results showed that proliferation inhibition in miR-485-5p@SPIONs-HuGSCs gradually increased 24 hours after microRNA@SPIONs were transfected into cells, and this was significantly higher than miR-mut@SPIONs-HuGSCs (Figure 3E). The experimental results showed that miR-485-5p@SPIONs significantly inhibit the *in vitro* proliferation, invasion, angiogenesis, and resistance towards chemotherapeutic agents in HuGSCs.

### miR-485-5p@SPIONs significantly inhibit the *in vivo* tumourigenicity of HuGSCs

**Fig 4:**
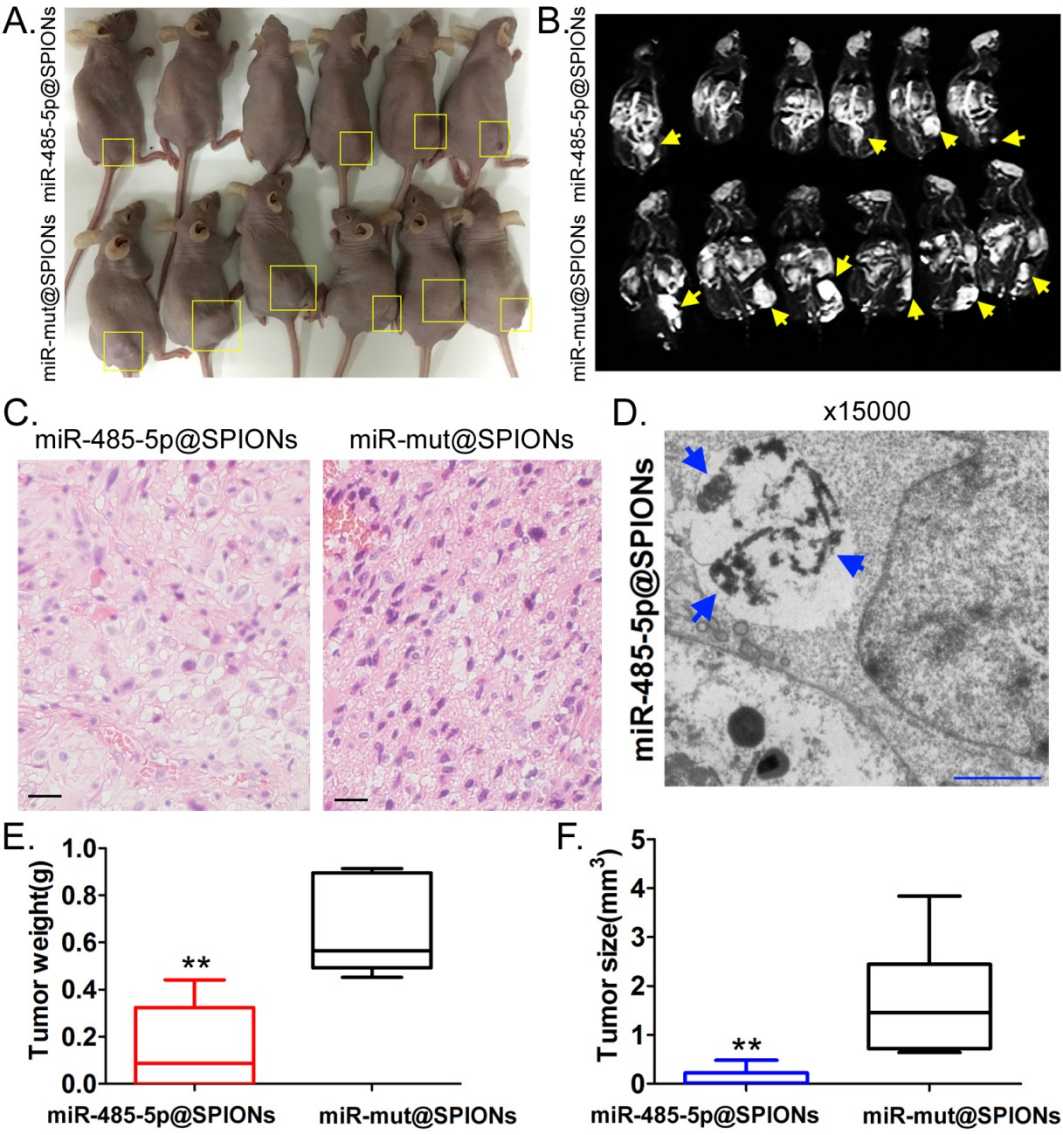
miR-485-5p@SPIONS inhibit the *in vivo* tumourigenicity of human glioma stem cells (HuGSCs). (A) Comparison of tumours formed on the backs of nude mice. (B) Magnetic resonance imaging results showed significant high-intensity signals at tumours on the backs of nude mice, suggesting the presence of metallic substances. (C) H&E staining results showed that the degree of malignancy of tumours originating from miR-mut@SPIONs-HuGSCs was significantly higher than that of those originating from miR-485-5p@SPIONs-HuGSCs. Scale bar = 30μm. (D) Transmission electron microscopy results showed that high-density electron clouds of aggregation of spherical particles were present in tumour cells. (E) The weight of tumours originating from miR-485-5p@SPIONs-HuGSCs was significantly lower than that of those originating from miR-mut@SPIONs-HuGSCs.**p<0.01 vs. miR-mut@SPIONs-HuGSCs group. t test. n=6. (F) The volume of tumours originating from miR-485-5p@SPIONs-HuGSCs was significantly lower than those originating from miR-mut@SPIONs-HuGSCs.**p<0.01 vs. miR-mut@SPIONs-HuGSCs group. t test. n=6.

Two groups of cells (miR-485-5p@SPIONs and miR-mut@SPIONs) were inoculated in the backs of nude mice. Nude mice were then euthanised at around eight weeks. We found that most of the nude mice that were inoculated with miR-mut@SPIONs-HuGSCs had larger tumours (Figure 4A). In contrast, only a few nude mice that were inoculated with miR-485-5p@SPIONs-HuGSCs had small tumours on their backs (Figure 4A). *In vivo* magnetic resonance imaging results showed significant high-intensity signals at tumours on the backs of nude mice, suggesting the presence of metallic substances (Figure 4B). Following that, tumour tissues were isolated and embedded in paraffin. Haematoxylin and eosin (H&E) staining results showed that the tumours originating from miR-mut@SPIONs-HuGSCs had larger nuclei and deeper staining, and had more and disorderly karyokinesis, suggesting that their degree of malignancy was significantly higher than that of those originating from miR-485-5p@SPIONs-HuGSCs (Figure 4C). Transmission electron microscopy results showed that high-density electron clouds of the aggregation of spherical particles were present in tumour cells, suggesting the presence of metallic nanoparticles (Figure 4D). In addition, tumour volume and weight measurement results also showed that both the volume and weight of tumours originating from miR-485-5p@SPIONs-HuGSCs were significantly lower than that of those originating from miR-mut@SPIONs-HuGSCs (Figures 4E, F). The above results showed that miR-485-5p@SPIONs significantly inhibitedtumourigenicityin HuGSCs.

### Tie1 activates FGF2 to promote the protein expression of GDNF/GFAP/BDNF

**Fig 5:**
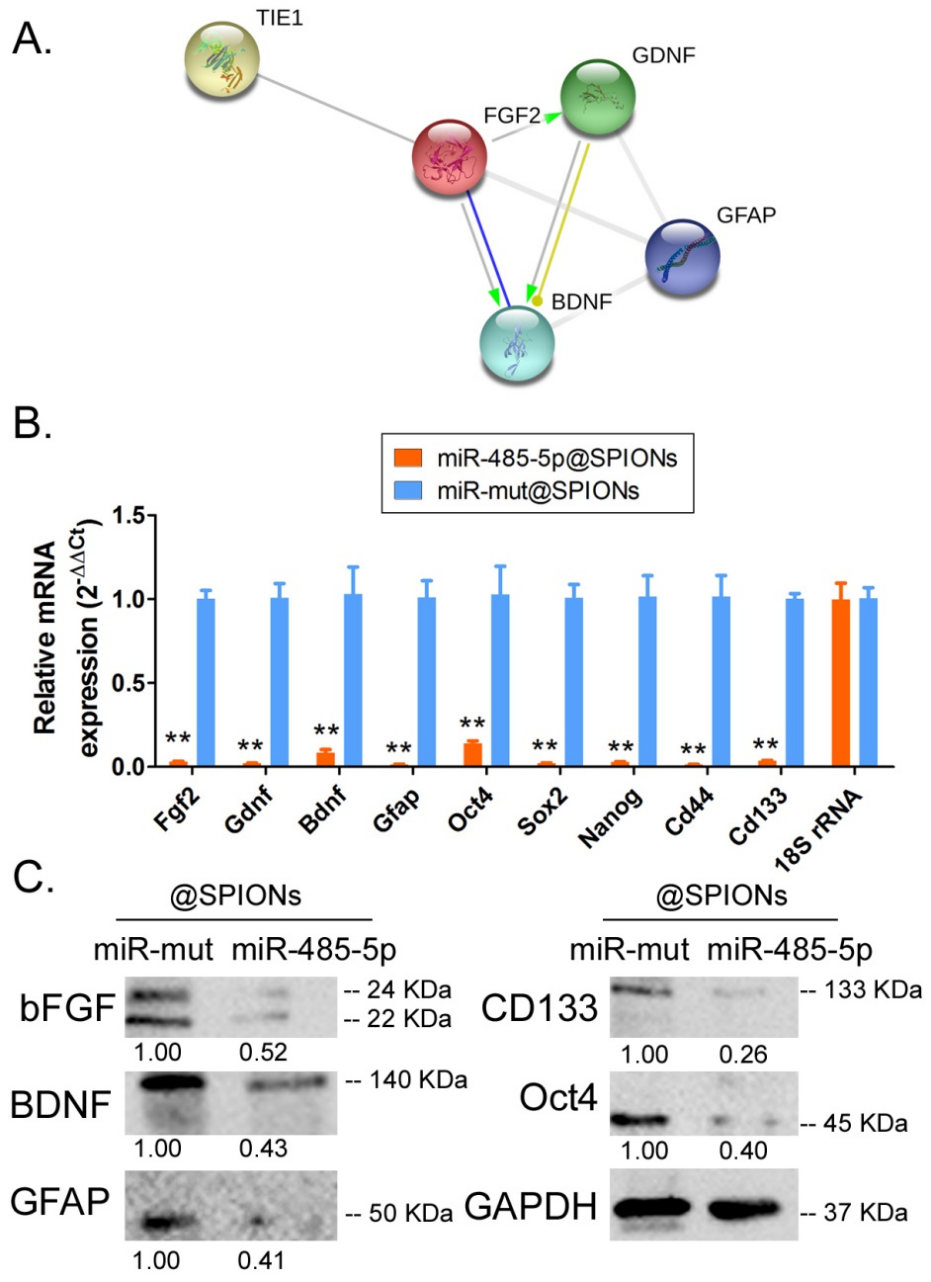
Tie1 activates FGF2 to promote the protein expression of GDNF/GFAP/BDNF. (A) Protein-protein interaction prediction software showed that FGF2 protein is downstream of Tie1 protein and FGF2 directly interacts with GDNF/GFAP/BDNF proteins. (B) qPCR results showed that the expression levels of stem cell markers and FGF2, GDNF, GFAP, and BDNF were significantly lower in miR-485-5p@SPIONs-HuGSCs. **p<0.01 vs. miR-mut@SPIONs-HuGSCs group. t test. n=3. (C) Western blot results showed that the protein expression levels of stem cell markers and FGF2, GDNF, GFAP, and BDNF were significantly lower in miR-485-5p@SPIONs-HuGSCs than in miR-mut@SPIONs-HuGSCs. **p<0.01 vs. miR-mut@SPIONs-HuGSCs group. t test. n=3.

Protein-protein interaction prediction software (STRING Version:11.0; https://string-db.org) was used to examine the downstream protein signalling network of Tie1. Software prediction results showed that the FGF2 protein is downstream of the Tie1 protein and FGF2 directly interacts with GDNF/GFAP/BDNF proteins (Figure 5A). Subsequently, qPCR and western blot results showed that in miR-485-5p@SPIONs-HuGSCs, the expression levels of stem cell markers (Oct4, Sox2, Nanog, CD44, and CD133), and FGF2, BDNF, GDNF, and GFAP were significantly lower thanlevelsin miR-mut@SPIONs-HuGSCs (Figures 5B, C). The results showed that miR-485-5p overexpression inhibits endogenous Tie1 expression, which inhibits the protein expression of GDNF/GFAP/BDNF by downregulating FGF2 expression.

**Fig 6:**
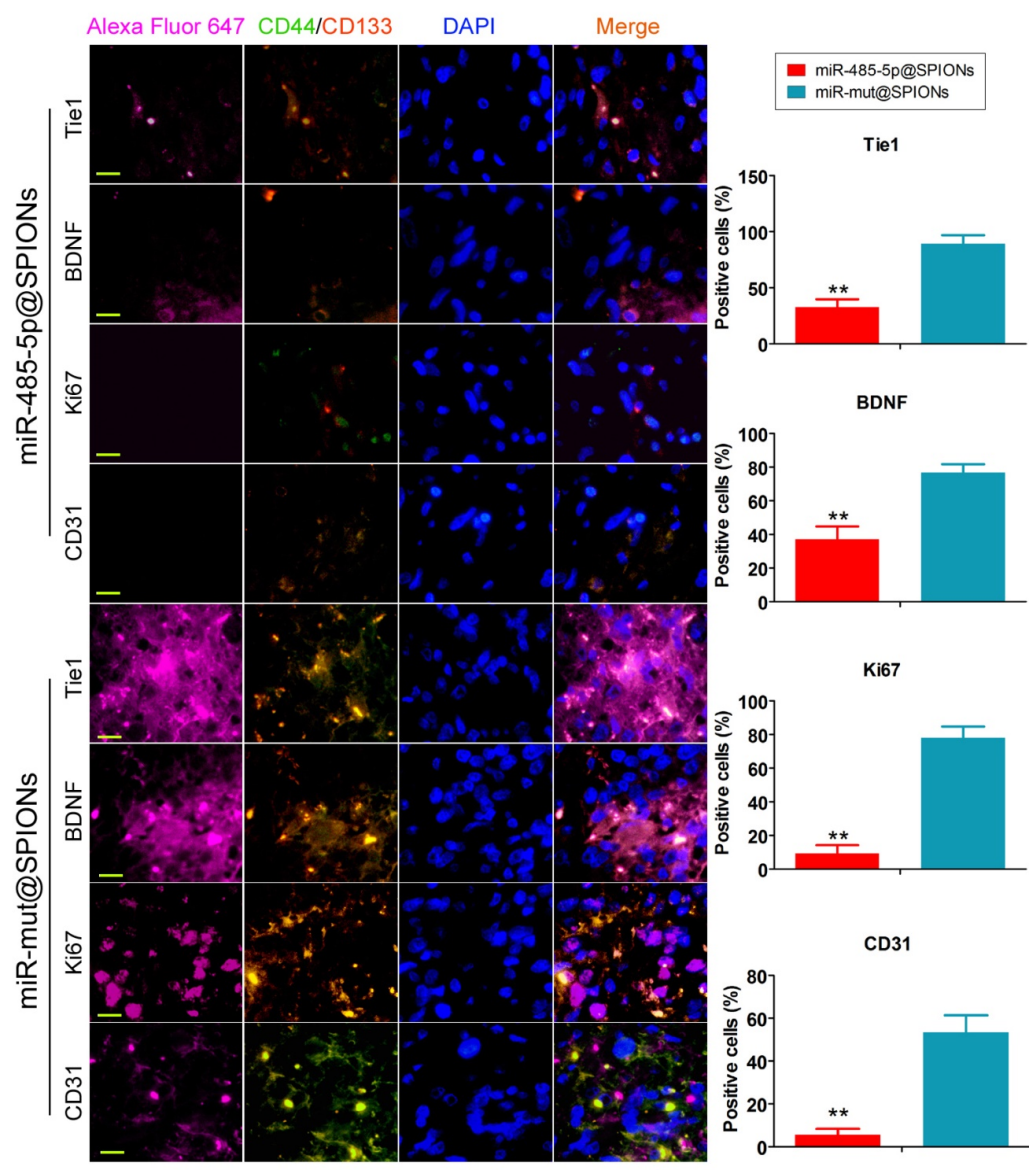
Low expression of proliferation and angiogenic proteins in tumours originating from miR-485-5p@SPIONs-HuGSCs. The proliferation, neurotrophic factors, and angiogenesis of glioma cells is directly proportional to Tie1 expression level and the number of cancer stem cells. When the number of cancer stem cells in tumour tissues is decreased and Tie1 expression is reduced, cell division and angiogenesis rates will decrease. **p<0.01 vs. miR-mut@SPIONs-HuGSCs group. t test. n=3. Scale bar = 30μm.

In addition, we employed immunofluorescence staining to verify the above phenomenon in tumour tissues. The experiment results showed that the number of tumour stem cells (proportion of CD44+/CD133+ cells) and Tie1-positive cells in tumours originating from miR-485-5p@SPIONs-HuGSCs was significantly lower than that in those originating from miR-mut@SPIONs-HuGSCs (Figure 6). At the same time, the proportion of cells that were positive for nuclear proliferation factor (Ki67), brain-derived neurotrophic factor (BDNF), and tumour angiogenesis marker (CD31) in tumours originating from miR-485-5p@SPIONs-HuGSCs were significantly lower than that of those originating from miR-mut@SPIONs-HuGSCs (Figure 6). The results showed that the proliferation, neurotrophic factors, and angiogenesis of glioma cells were directly proportional to the Tie1 expression level and number of cancer stem cells. When the number of cancer stem cells in tumour tissues is decreased and Tie1 expression is reduced, cell division and angiogenesis rates will decrease.

## Discussion

Currently, more and more studies have shown that SPIONs are good nanomaterials. SPIONs have the following unique advantages: (1) SPIONs have good biocompatibility as biomaterials; (2) The surfaces of SPION particles can be modified with different substances, thereby increasing the stability of nanoparticles and preventing recognition by the immune system. Therefore, SPIONs have ideal surface structures; (3) SPIONs have unique magnetic responses and can be used in magnetic targeting and magnetic thermal therapy; (4) SPIONs are ideal magnetic resonance imaging contrast agents and are used in clinical practice forimaging liver cancers and lymph node metastases; (5) Targeting: SPIONs combined with an external magnetic field can directly and accurately deliver drugs into target sites for the slow and controlled release of drugs. Therefore, SPIONs have significantly targeted carrier functions 22-26. Regarding cancer treatment, Kobayashi et al. developed magnetoliposomes to treat various types of cancers, such as brain tumours, tongue cancer, gastric cancer, and melanoma 27. In order to increase the affinity of SPIONs towards tumour cells (which are negatively charged), the researchers modified SPIONs with cationic liposomes to obtain cationicmagnetoliposomes. At the same time, cationicmagnetoliposomes were directly injected into the tumour site to avoid adsorption by normal cells. They found that cationicmagnetoliposomes show good efficacy against the various tumours studied 27. In previous studies, liposome carriers are often used to transfect plasmid DNA due to the lipid bilayer structure of eukaryotic cells. Although this technique has been used for many years, liposomes possess a certain degree of toxicity, and transfection efficiency is not ideal 6, 7, 18, 28. As research into nanomaterials deepens, some studies have reported using nanoparticles as a medium for drugs or nucleic acid transfection 7, 18-20, 22, 23, 26, 29. We previously found that SPIONs can be loaded with microRNA-374a for overexpression in gliomas, thereby inhibiting the expression of endogenous NRN1 and inhibiting glioma proliferation, invasion, and tumourigenicity in nude mice 7. At the same time, siRNA carried by SPIONs can efficiently inhibit the expression of the target gene HOTAIR and remove its inhibition against PDCD4 transcription, thereby reducing glioma proliferation and invasion 6. In addition, we also found that miR-326@SPION can efficiently inhibit the expression of the GPR91/STAT3/VEGF signalling pathway and weaken the *in vivo* and *in vitro* viability of endometrial cancer stem cells 6. These studies all demonstrated the advantages of SPIONs as nucleic acid carriers. Also, SPIONs can be enriched at tumour sites, so that the iron oxide content of the tumour site is relatively higher than that of other healthy tissues. Iron oxide appears as shadows during radioactive imaging, which can be used to locate tumour tissues and distinguish them from healthy tissues, and carries important significance in tumour diagnosis 30. Schleich et al. found that PLGA-modified SPIONs have significant effects in breast cancer diagnosis. Their study showed that PLGA-modified SPIONs not only act as drug delivery vectors, but also play a diagnostic role in magnetic resonance imaging 25. In addition, SPIONs that are combined with certain biological ligands can be used for targeted tracing and imaging. For example, c(RGD-fK) peptide-modified SPIONs can bind to integrin αvβ3 and achieve targeted imaging of tumours *in vivo*. After intravenous injection, the modified SPIONs will first accumulate at the tumour site that has high integrin αvβ3 expression. Following that, magnetic resonance imaging can be used for tracing 31. Usually, SPION-derived MRI contrast agents may be classified into two categories based on particle size: >50-nm SPIONs and <50-nm SPIONs 32. SPIONs are usually used in magnetic resonance imaging (MRI) of the liver, spleen, and lymph nodes. Since SPIONs tend to be recognised and phagocytosed by reticuloendothelial cells in the liver after they have entered the body, the T2-weighted signal of healthy tissues is weakened in MRI and appears black or dark in colour. Due to the absence of the ability to phagocytose SPIONs in lesion sites in the liver, the T2-weighted signal in MRI remains unchanged 32. SPIONs are often used as contrast agents in brain tumours, blood vessels, and blood pools. Since they can avoid regulation by the reticuloendothelial system, SPIONs can circulate in the bloodstream for long periods of time 32. Therefore, the utilisation of the dual effects of SPIONs in imaging and targeted therapy has significant potential for targeted tumour treatments.

This study was the first to demonstrate the function of Tie1 in gliomas. Tie1 is often studied in the occurrence of vascular development and cardiovascular diseases 10-17, At present, there are no reports on its relationship with glioma, particularly glioma stem cell viability. However, we employed bioinformatics software for predicting protein-protein interactions and found that Tie1 can interact with some cellular factors, particularly nerve growth factors such as BDNF, GDNF, and GFAP. The results were extremely interesting. This is because studies have reported that BDNF, GDNF, and GFAP are intimately associated with gliomas as well as tumourigenicity, progression, and prognosis, and high levels of BDNF, GDNF, and GFAP can significantly promote glioma viability 4, 5, 33. When bioinformatics prediction found that Tie1 is associated with the aforementioned nerve growth factors, we believed that Tie1 should have positive regulatory effects on gliomas (stem cells). At the same time, we hypothesised that Tie1 expression levels will affect the expression of downstream BDNF, GDNF, and GFAP. The experimental results showed that our hypothesis was correct: After miR-485-5p@SPIONs were overexpressed in HuGSCs, the expression of both nerve growth factors (BDNF, GDNF, and GFAP) and stemness factors (Oct4, Sox2, and Nanog) all significantly decreased. At the same time, the *in vivo* and *in vitro* viability of glioma stem cells were significantly inhibited. Hence, we can confirm that Tie1 is an important factor that maintains glioma stem cell activity. Of course, there are still many research directions that can be expanded in this study. For example, SPIONs can also kill tumor cells by themselves 34-36. It has been found that SPIONs can kill tumor cells in animal models through magnetic induction of nano thermal effect, and play a therapeutic role. At the same time, SPIONs have been proved to have some biological toxicity 37. In practical clinical application, it is of great significance to explore a reasonable dose concentration and time cycle for improving the therapeutic effect of SPIONs and reducing its own biological toxicity. In addition, although we report the epigenetic mechanism of microRNA regulating Tie1 expression, how to translate it into clinical therapy is still worth further exploring.

In conclusion, this study showed that SPIONs drive the overexpression of miR-485-5p in human glioma stem cells and efficiently inhibit Tie1 expression levels to significantly reduce the *in vivo* and *in vitro* viability of gliomas. Meanwhile, SPIONs is used to overcome intracellular and extracellular barriers, increase local microRNA concentration, and increase transfection efficiency. So, our research will provide the novel insights for the clinical translational medicine application of SPIONs.

## Figures and Tables

**Fig 7:**
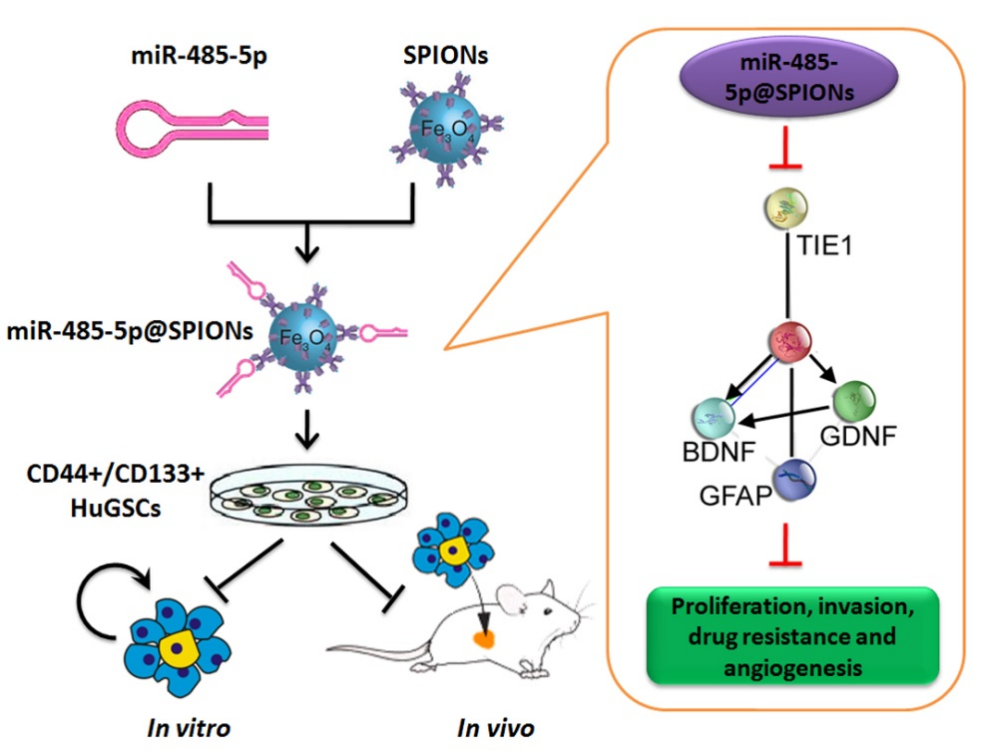
Effector mechanism by which SPIONs drive miR-485-5p inhibition of human glioma stem cell (HuGSC) viability by silencing Tie1 expression.

**Table 1:** Antibodies' information

Antibodies	Companies	Applications
Rabbit anti-human TIE1 (#23111)	Cell Signaling Technology, Inc. (Danvers, MA, USA)	IF (1:100) WB(1:1000)
Rabbit anti-human BDNF (#3897)	Cell Signaling Technology, Inc. (Danvers, MA, USA)	IF (1:100) WB(1:1000)
Rabbit anti-human bFGF (#3196)	Cell Signaling Technology, Inc. (Danvers, MA, USA)	WB(1:1000)
Rabbit anti-human GFAP (#12389)	Cell Signaling Technology, Inc. (Danvers, MA, USA)	WB(1:1000)
Rabbit anti-human Ki67 (#9129)	Cell Signaling Technology, Inc. (Danvers, MA, USA)	IF (1:100)
Rabbit anti-human CD133 (#64326)	Cell Signaling Technology, Inc. (Danvers, MA, USA)	IF (1:100)
Rabbit anti-human CD31 (#77699)	Cell Signaling Technology, Inc. (Danvers, MA, USA)	IF (1:100)
Rabbit anti-human Oct4 (#2750)	Cell Signaling Technology, Inc. (Danvers, MA, USA)	WB(1:1000)
Rabbit anti-human GAPDH (#5174)	Cell Signaling Technology, Inc. (Danvers, MA, USA)	WB (1:1000)

## Abbreviation

SPIONs: Superparamagnetic iron oxide nanoparticles
HuGSCs: Human glioma stem cells
PEI: Polyethylenimine
qPCR: Quantitative real-time PCR
FBS: Fetal bovine serum
MTT: 3-(4,5-dimethylthiazol-2-yl)-2,5-diphenyltetrazolium bromide
PVDF: Polyvinylidene fluoride
TBST: Tris-buffered saline-Tween 20
ECL: Chemiluminescence
PI: Propidium iodide
DAPI: 4,6-diamidino-2-phenylindole
SE: Standard error
H&E staining: Haematoxylin and eosin staining
BDNF: Brain-derived neurotrophic factor
